# Calibration of confidence and assessed clinical skills competence in undergraduate paediatric OSCE scenarios: a mixed methods study

**DOI:** 10.1186/s12909-018-1318-8

**Published:** 2018-09-17

**Authors:** Dara O’Donoghue, Gail Davison, Laura-Jo Hanna, Ben McNaughten, Michael Stevenson, Andrew Thompson

**Affiliations:** 1 Royal Belfast Hospital for Sick Children, 180-184 Falls Road, Belfast, BT12 6BE UK; 2 Centre for Medical Education, Queen’s University, Whitla Medical Building, 97 Lisburn Road, Belfast, BT97AE UK

**Keywords:** Confidence, Competence, Skills, Calibration

## Abstract

**Background:**

The relationship between confidence and competence in clinical skills development is complex but important. This study aims to determine undergraduate paediatric student confidence in performing three common paediatric clinical skills framed as Objective Structured Clinical Examination (OSCE) scenarios and to compare this with subsequent assessed performance. The study also aims to explore possible barriers to successful paediatric skills completion.

**Methods:**

A mixed-methods study was conducted on medical students. Cross-sectional questionnaire data relating to confidence in performing a number of paediatric skills were compared with assessed paediatric skills competency. Focus groups were carried out to identify themes in paediatric skills completion to triangulate this data.

**Results:**

Eighty-five medical students participated in the study. Students had high levels of pre-test confidence in their ability to perform paediatrics skills. However agreement between pre-test confidence and subsequent task performance was poor and students had significantly greater belief in their skills ability than was subsequently demonstrated. Focus groups identified paediatric skills complexity, conflicting teaching and having limited supervised skills opportunities and as being possible contributory factors to this discrepancy.

**Conclusions:**

Student paediatric skills confidence is not matched by performance. The reasons for this are diverse but mostly modifiable. A major factor is the lack of supervised skills experience with appropriate feedback to support students in learning to calibrate their confidence against their competence. A number of recommendations are made including the introduction of formative assessment opportunities.

## Background

The relationship between a practitioner’s confidence in performing a skill and subsequent performance is intriguing and important. Confidence in performing a task influences the willingness and ultimate decision to undertake the task [1, 2]. However, if the confidence does not match the subsequent performance, the practitioner may fall short and potentially risk an adverse incident. Moreover, level of experience correlates well with confidence but not with assessed performance [3, 4]. A recent study of medical student prescribing skills found a poor correlation between reported confidence and actual competence and concluded that students lack insight into their strengths and weaknesses in prescribing [5]. It is therefore important that confidence and competence are not used synonymously [2].

Tomorrow’s Doctors (2009) has been used as the roadmap for undergraduate medical training in the United Kingdom [6]. This General Medical Council (GMC) document outlines the expected competencies of a newly qualified doctor. Among these specific competencies are drug and intravenous fluid (IVF) prescribing and plotting of growth charts. These have been completed poorly in Queens University Belfast (QUB) undergraduate paediatric Objective Structured Clinical Examinations (OSCEs) over the last five years (personal communication with QUB examiners).

In the Undergraduate medical course at QUB students start adult clinical skills training in their second year. Their exposure to paediatric medicine does not occur until the ‘Healthcare of Children’ module which is a six week course in the fourth year of their degree (the module runs six times through the academic year with a total of approximately two hundred and forty students per year). A week of core teaching (lectures, practical sessions and online learning) precedes a five week clinical attachment in a paediatric ward. Learning outcomes are mapped to a paediatric syllabus. The syllabus lists clinical skills that students are expected to have completed by the end of the module. Students have access to online lectures on fluid prescribing and completion of growth charts. It is a mandatory requirement that all students complete the online British Medical Journal (BMJ) module on fluid prescribing [7]. During the clinical attachment students also attend a drug prescribing workshop where they practice completing drug prescribing records.

Due to their limited exposure to paediatrics compared with adult medicine, student confidence in the performance of paediatric clinical skills may be expected to be at a low level. The relationship between medical student confidence and competence in the performance of core paediatric clinical skills has not previously been explored.

The aim of this study is to assess undergraduate paediatric student confidence in performing three common and important clinical paediatric skills, compare this with actual performance and determine any barriers to successful skill completion.

## Methods

### Study design

A mixed methods study of fourth year medical students was conducted. Medical student pre-test confidence in completing common paediatric skills was determined using a questionnaire. This was compared with skills competence using a skills assessment. Focus groups were conducted to explore the relationship between these levels of confidence and assessed competence.

### Setting

A convenience sample of Fourth year medical students at QUB in the first two modules of the academic year 2014–15 were invited to participate in the study in seven paediatric units during the final week of the ‘Healthcare of Children’ module. Students were informed that this was not part of the module and was voluntary, anonymous and inconsequential. Written consent from the students was obtained. Ethical approval was granted by the QUB School of Medicine and Dentistry Research Ethics Committee.

### Quantitative methods

Each student completed a two-part comparative evaluation which included a paediatric skills questionnaire and a subsequent assessment of performed skills. The questionnaire was constructed by the researchers to determine levels of confidence in performing mandatory skills listed in the syllabus. These skills need to have been completed for successful completion of the module. On a four point Likert scale (1 = Excellent, 2 = Good, 3 = Pass 4 = Fail) students documented their anticipated skills performance. The skills included ‘prescribing common paediatric medication’, ‘prescribing paediatric IVF’ and ‘plotting growth parameters on an appropriate growth chart’. The questions were reviewed and refined by an iterative process involving all the researchers ensuring content validity.

Skills-based scenarios were developed by the researchers based on previously standardised paediatric OSCE stations. These had been standard-set using the Angoff method. The scenarios assessed the three skills from the questionnaire - medication prescribing, plotting of growth parameters on growth charts and IVF prescribing. They were completed on the regional Paediatric Medicine Prescription and Administration Record, the UK-WHO Growth Chart 0–4 years and the regional Paediatric Daily Fluid Prescription and Balance Chart [8, 9]. Students were awarded a global score based on QUB OSCE assessment guidance [10]. This assessment stage was initiated after all survey forms had been collected. Students were given ten minutes to complete each stage and were unable to modify any completed documentation after submission. All charts were original and numbered in advance for anonymised identification. Students were provided with a British National Formulary for Children (BNFC) to assist prescribing [11]. The skills were assessed by two examiners (GD and LH) using the modified global scoring system as being: 1 = Excellent, 2 = Good, 3 = Pass 4 = Fail [10].

Data were analysed using IBM SPSS Statistics version 20.0 (SPSS Inc. Armonk, New York). The Internal consistency of the questionnaire was evaluated using the Crohnbach’s alpha coefficient and the Kuder- Richardson formula 20 (KR_20_) was used to determine internal consistency in relation to passing or failing the skills assessments. Paired confidence and assessed skills competence results were compared using Wilcoxon signed ranks exact probability tests and agreement between confidence and assessed competence was analysed using the linear- weighted Kappa test. The kappa statistic is a measure of agreement standardised to lie between − 1 and 1 where 1 indicates perfect agreement, 0 is what would be expected by chance and − 1 indicates potential disagreement. Statistical significance was assumed for *p* values < 0.05.

### Qualitative methods

Focus groups were used to gain a deeper understanding of student skills performance. An e-mail inviting participation was sent to students who had completed the skills assessment. Written consent was obtained from volunteers.

The focus groups were moderated (DOD) to encourage guided discussion. To facilitate moderation, key themes identified from the skills assessment were used to create a focus group guide. These themes were extracted following independent review of the skills data by three authors (DOD, AT and BM). Focus group discussions were recorded with two devices and transcribed verbatim. The authors reviewed the transcripts independently and identified preliminary codes. A framework analysis technique was used to identify emerging themes for each skill [12, 13]. The number of focus groups to be held was determined by the stage at which this iterative process had reached thematic saturation with a minimum number of two focus groups.

## Results

The student consent rate to participate in the questionnaire and skills assessment study was 100% (85 students out of 85). All students completed both the questionnaire and three skills assessments. The Crohnbach’s alpha coefficient for the questionnaire data was 0.81 suggesting high levels of internal consistency. The skills assessments also demonstrate high levels of internal consistency (KR_20_ values for drug prescribing, growth chart plotting, and IV fluid prescribing were 0.70, 0.89 and 0.89 respectively).

The cross-tabulations for student confidence ratings and skills assessment scores for each skill are shown in Table 1.

**Table 1:** Cross-tabulations of pre-test skills confidence ratings (1–4) against skills assessment scores (1–4) for (a) Drug prescribing (b) Growth plotting and (c) IVF prescribing

(a)					
Drug prescribing	Skills assessment score				
Student confidence rating		1	2	3	4
	1	1	26	42	6
	2	0	0	1	2
	3	0	1	5	1
	4	0	0	0	0
(b)					
Growth plotting	Skills assessment score				
Student confidence rating		1	2	3	4
	1	0	2	13	21
	2	0	0	0	1
	3	0	1	3	19
	4	0	0	11	14
(c)					
IVF prescribing	Skills assessment score				
Student confidence rating		1	2	3	4
	1	3	18	16	5
	2	0	18	18	3
	3	0	4	0	0
	4	0	0	0	0

For each of the assessed skills tasks, there was little or no agreement between pre-test confidence and assessed competence (Tables 1 and 2).

**Table 2:** Agreement for pre-test skills confidence ratings and skills assessment scores

	Kappa with linear weights	95% Confidence Intervals	
Medication	- 0.01	- 0.07	0.05
Growth Chart	- 0.02	- 0.10	0.05
IV Maintenance fluids	0.02	- 0.01	0.05

**Fig. 1:**
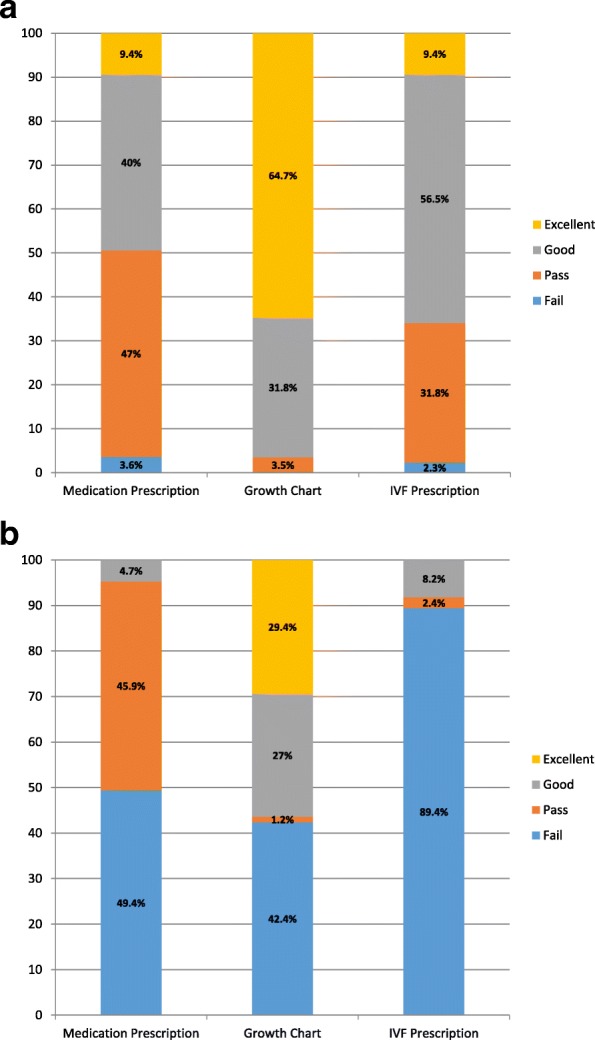
Bar charts representing (**a**) Students’ pre-test confidence (**b**) Students’ skills assessment

The pre-test skills confidence and assessed skills assessment scores are represented graphically in Fig. 1a and Fig. 1b respectively. The students’ confidence in their ability to successfully complete the task was not matched by their task competence with students believing that they would perform to a higher level than they actually did (Wilcoxon signed ranks exact probability tests for paired comparisons of confidence and competence for each of the three skills - *p*-value < 0.001).

### Focus groups

Twenty six students volunteered to take part in focus groups and students were assigned to focus groups in the order that they volunteered. There were six students in each focus group and thematic saturation occurred following completion of three focus groups. Analysis of focus group transcripts identified five distinct overarching themes. The themes were similar for the three tasks (Table 3).

**Table 3:** Themes emergent from analysis of each Focus Group (FG)

Skill	Overarching themes	FG1	FG2	FG3
Fluid prescribing	Conflicting teaching	x	x	
	Complexity		x	x
	Prior experience	x		x
	Prior supervised experience	x	x	x
	Chart familiarity	x	x	x
Drug prescribing	Complexity	x	x	x
	Prior experience	x	x	x
	Prior supervised experience	x		x
	Chart familiarity	X	x	x
Growth Charts	Complexity	x	x	
	Prior experience	x	x	x
	Prior supervised experience		x	x
	Chart familiarity	x	x	x

Five themes were identified and a summary of sub-themes with illustrative student quotes within each is provided below:

#### Conflicting teaching

This theme only arose for fluid prescribing. Teaching on IVF prescribing varied depending on which hospital the student had been based in. The advice on when and when not to prescribe potassium in maintenance fluids created the most difficulty:

‘In Hospital A I had been taught not to prescribe potassium chloride for maintenance fluids without an electrolyte profile being available but now I realise this is completely different to what students has been told in Hospital B.’

Prescribing potassium in IV fluids for a child whose blood electrolyte profile is not known is potentially dangerous as the potassium levels can rise and potentially result in an abnormality of the heart rhythm. Therefore such conflicting advice may lead to a serious adverse incident.

#### Complexity

It became apparent that students had limited experience of drug or IVF prescribing in previous adult-medicine attachments. In addition, students felt that incorporation of a weight calculation in paediatric drug and IVF prescribing significantly increased the task complexity compared to completion of the same task in adult medicine. Many students described how the completion of these paediatric skills was more challenging because of this. Correcting for prematurity in plotting growth was unfamiliar and confusing to some students:

‘It’s ok plotting a baby’s height and weight but plotting a premature baby is confusing because I don’t know whether to correct to 38 weeks or 40 weeks.

Correctly plotting and tracking a premature baby’s growth parameters accurately by taking account of the degree of prematurity (rather than simply plotting their chronological age) is extremely important in deciding whether a baby is achieving appropriate weight gain and brain growth for their age.

#### Prior experience

There was variation in the experience and opportunities that students had in skills completion. All students had completed a drug prescribing workshop. They had also completed the required number of patient admissions. As part of the documentation for admitting patients to a ward students are asked to plot each patient’s height and weight on an appropriate growth chart. It is assumed that completion of a growth chart is done at this time. However students admitted that they occasionally forgot to do this. Students had viewed an online lecture on fluid prescribing and all had completed an online BMJ module on paediatric fluid prescribing but some had not previously completed an actual fluid prescription:

‘I had seen the online lecture that takes us through fluids step-by step so I was happy that I knew what to do. When I was faced with the fluid chart and had to do it then it was a different matter’.

This quotation illustrates the point that, for some students, there was a clear gap between theory and application. They appeared to assume that, armed with the relevant background theoretical knowledge, the clinical skill would be straightforward. The skills assessment demonstrated that this is not necessarily the case.

#### Prior supervised experience

Students differentiated between skills experience and supervised skills experience. Many students had completed fluid prescriptions but rarely received feedback on this to allow them to calibrate their skills. Most students had plotted growth charts when admitting patients to a ward but these were not always appraised by a supervising doctor:

‘I try to complete the growth chart for every child I see as told. The doctors that I present my cases to go through the diagnosis and tests but don’t look over my notes or the photocopied growth chart I have filled in.’

The students appeared concerned that they do not always receive optimal supervision or specific feedback tailored to their learning needs.

#### Chart familiarity

A number of students had prescribed a medication correctly but in the wrong section of the drug prescribing record e.g. an oral antibiotic in the intravenous section and they appeared to be unfamiliar with navigating the prescription record. A few students also failed to complete the record of drug allergies:

‘I was focusing on getting the right dose written in the right place. I hadn’t filled in drug allergy boxes previously and I didn’t notice it when looking where to write the prescription’.

It became apparent that there were slight variations in the drug prescribing charts between the paediatric units where students were based for their paediatric attachment. To standardise prescribing practice a national paediatric drug prescribing record has subsequently been developed and is now used in all paediatric units in Northern Ireland.

## Discussion

This study shows relatively poor skills performance for all three assessed tasks. As all students had received instruction and had experience in completing most or all of these tasks, the results are concerning. There is no agreement between confidence and subsequent performance of a paediatric skill. These results align with those of a postgraduate study of newly-qualified doctors that found poor correlation between self-reported confidence and assessed competence in the completion of seven clinical skills [14]. The focus group data identified a number of potentially modifiable factors related to skills performance. This is the first study to explore this relationship in paediatric clinical OSCE scenarios and to triangulate confidence and competence data using qualitative methodology.

The 4th year paediatric attachment is likely to be the last contact with paediatric medicine until a student becomes a Foundation Year 2 (FY2) doctor in paediatrics. Therefore it is important that this opportunity is optimised. Monitoring of growth is an essential component of childhood healthcare and the Department of Health (DOH) and Royal College of Paediatrics and Child Health (RCPCH) recommend that professionals who plot or interpret UK-WHO growth charts should receive suitable training or supervision [15]. However, growth charts are frequently missing from clinical notes or are incomplete [16–20]. A case review of the clinical notes of fifty hospitalised infants found that nearly 30 % of points plotted on infant growth charts were plotted in error [21]. The main sources of error identified were in plotting age and correcting for prematurity. In a further study, up to 20 % of health-care professionals were unable to interpret the UK-WHO growth charts with many receiving no formal teaching in their use [22]. In the present study performance was better for completion of growth charts than for the prescribing tasks. This may be as a result of having relatively more supervised practice in plotting, as students are expected to complete and present fifteen patient records. A completed growth chart should be included as part of this. However this was occasionally omitted and the focus groups revealed that, even when completed, some students reported that they were not always reviewed by a supervising doctor. The focus groups also echoed the results of a previous study in finding that completion of growth charts for ex-preterm infants was particularly difficult [21]. Correcting for prematurity was problematic and many students stated that they had not specifically done this before.

In a systematic literature review of the level of clinical preparedness in acute care, United Kingdom graduates feel poorly prepared in the area of medication prescribing [23]. Prescribing errors have been found in 8.4% and 10.3% of prescriptions completed by Foundation Year 1 and 2 doctors respectively [24]. Despite this, much of the teaching of paediatric prescribing in the postgraduate domain has been by lectures with little assessment of competency [25]. Interestingly, a recent review of strategies to ensure new graduates are safe prescribers suggests that prescribing teaching should focus on the development of expertise rather than competency [26]. The development of a ‘theoretical framework of knowledge application’ advocates that prescribing should be contextualised and embedded within the workplace setting rather than being studied in isolation. It has been shown that practical prescribing courses delivered to undergraduate medical students improve postgraduate prescribing [27]. Safe and competent prescribing should be an aim of educators charged with developing the undergraduate curriculum.

Prescribing intravenous fluids is an important skill for all hospital doctors. Paediatric fluid prescribing is complicated by the physiological and anatomical differences between adults and children, thus making it a greater challenge in this population. The National Institute of Clinical Excellence (NICE) has recently produced guidelines specific to children [28]. Prescribing the wrong type of fluid to children can result in significant morbidity and in some cases death [29]. A recent high-profile public inquiry into childhood deaths related to hyponatraemia in Northern Ireland has highlighted the importance of fluid prescribing [30]. Consequently the government released guidance on fluid prescribing in children [31]. Out of the three simulated tasks, students were very confident about successfully completing this task but actually performed least well overall. It became apparent in the focus groups that some students had received conflicting teaching during their clinical attachments. This has also been shown in a recent study examining the challenges of fluid prescribing [32]. As a result of this study medical students have developed fluid prescribing vignettes that have been disseminated regionally to ensure uniformity in teaching. In the focus groups students specifically describe the limited skills supervision received in fluid prescribing. The Regional Hyponatraemia Competence Framework document advocates ‘demonstrated competency’ in the safe prescription of intravenous fluids to avoid the risk of hyponatraemia [33]. This involves supervised prescribing and completion of case studies ratified by a senior member of medical staff. Extrapolating this advice to student intravenous fluid prescribing would suggest that students should also demonstrate competency and this may help them to calibrate their progress in skills development. This ratification could be by a senior doctor although a recent study suggests that medical students prefer ‘near-peer’ education led by supervised junior medical staff [34]. In addition, the use of peer-assisted learning may help to develop paediatric knowledge and skills [35]. Although the benefit of timely and specific feedback is well recognised, and students are keen to receive it, a recent study exploring feedback given to medical students on their communications skills suggests that it is uncommon for students to be directly observed practicing this skill and even less common to receive feedback on it [36].

It is recognised that newly qualified doctors have deficiencies in clinical skills [37]. Preparedness for practice and induction courses have been introduced for Foundation doctors to aid this transition and help with skills acquisition. However, Foundation Year 1 (FY1) doctors do not work in Paediatric wards and the paediatric content of these Foundation courses is minimal [38, 39]. Paediatrics is a competency-based specialty and in the UK the curriculum for postgraduate paediatric training, published by the RCPCH, is modelled on competencies that need to be achieved for successful progression [40]. It is anticipated that completion of these competencies will ultimately lead to better patient outcomes. The RCPCH has recently published a competency-based undergraduate curriculum that specifies essential paediatric skills required of a medical student [41]. The curriculum includes the three skills examined in this study. We propose that acquisition of the skills listed in the curriculum is ratified by demonstration of competence in the clinical setting analogous to Directly Observed Procedural Skills (DOPs) in the postgraduate domain [42].

Entrustable Professional Activities (EPAs) are units of professional activity that can be entrusted to a competent learner and comprise of a number of core competencies. They have been adopted in postgraduate competency-based frameworks and incorporate work-based learning, trust and transparency. They are increasingly being used in undergraduate training to monitor and ratify skills completion and core EPAs for entering a residency programme were proposed and piloted by the Association of American Colleges (AAMC) in 2014 [43]. EPAs will likely help with the integration of postgraduate and undergraduate training and have a positive impact on the development of undergraduate medical skills training.

Although there are important messages to drawn from this study, there are also a number of limitations. It is a single-centre study involving a convenience sample of students so it is not known if the results are applicable to other centres. The examiners conducting the skills assessments made an agreed assessment following a period of discussion. The examiners were experienced in examining OSCE scenarios but it is recognised that having agreed decisions rather than analysing independent assessments may contribute to differences. It was fortunate that the study was able to get full participation from all students approached. There were no penalties for opting out of the study. It was clear from focus group discussions that students were keen to get examination practice and that this was likely the reason for full participation.

## Conclusion

Skills proficiency can only be acquired through practice. However, practice without appropriate feedback may result in high levels of confidence without resulting in high levels of skills competence. This study has shown that, for completion of three important paediatric tasks, student confidence does not align with competence. These findings are similar to studies comparing confidence and skills competence in adult medicine. An overly confident practitioner who is unable to successfully complete these fundamental paediatric tasks is potentially unsafe. Regarding confidence in skills completion, the focus should move towards the development of trainee task-oriented confidence or self-efficacy rather than the isolated non-specific trait of confidence alone. The study identified modifiable factors that should be addressed to improve skills acquisition and these may also help in the development of self-efficacy. Standardisation of teaching is essential. Demonstrated and ratified competence is required to ensure that students are capable of performing clinical skills. This allows students to apply knowledge, skills and attitudes in a safe, supportive environment. This process may be integrated into the workplace to allow students to calibrate their clinical skills development. The implementation of a comprehensive framework of supervised clinical skills taught and assessed in undergraduate paediatric training will be key to the development of safe and competent medical practitioners.

## Abbreviations

AAMC: Association of American Colleges
BMJ: British Medical Journal
BNFC: British National Formulary for Children
DOH: Department of Health
DOPS: Directly Observed Procedural Skills
FY1: Foundation Year 1
FY2: Foundation Year 2
GMC: General Medical Council
IVF: Intravenous fluids
KR20: Kuder-Richardson formula 20
NICE: National Institute of Clinical Excellence
OSCE: Objective Structured Clinical Examination
QUB: Queens University Belfast
RCPCH: Royal College of Paediatrics and Child Health
SLE: Supervised Learning Event
